# *Citrobacter freundii* fitness during bloodstream infection

**DOI:** 10.1038/s41598-018-30196-0

**Published:** 2018-08-07

**Authors:** Mark T. Anderson, Lindsay A. Mitchell, Lili Zhao, Harry L. T. Mobley

**Affiliations:** 1 University of Michigan Medical School, Department of Microbiology and Immunology, Ann Arbor, MI USA; 2 University of Michigan School of Public Health, Biostatistics Department, Ann Arbor, MI USA

## Abstract

Sepsis resulting from microbial colonization of the bloodstream is a serious health concern associated with high mortality rates. The objective of this study was to define the physiologic requirements of *Citrobacter freundii* in the bloodstream as a model for bacteremia caused by opportunistic Gram-negative pathogens. A genetic screen in a murine host identified 177 genes that contributed significantly to fitness, the majority of which were broadly classified as having metabolic or cellular maintenance functions. Among the pathways examined, the Tat protein secretion system conferred the single largest fitness contribution during competition infections and a putative Tat-secreted protein, SufI, was also identified as a fitness factor. Additional work was focused on identifying relevant metabolic pathways for bacteria in the bloodstream environment. Mutations that eliminated the use of glucose or mannitol as carbon sources *in vitro* resulted in loss of fitness in the murine model and similar results were obtained upon disruption of the cysteine biosynthetic pathway. Finally, the conservation of identified fitness factors was compared within a cohort of *Citrobacter* bloodstream isolates and between *Citrobacter* and *Serratia marcescens*, the results of which suggest the presence of conserved strategies for bacterial survival and replication in the bloodstream environment.

## Introduction

Septicemia is consistently among the 15 leading causes of all death in the United States, with greater than 40,000 deaths attributed in the most recently recorded year^1^. Gram-positive and Gram-negative bacterial species along with fungal pathogens are all contributors to the substantial healthcare burden associated with bloodstream infections (BSI) over the past several decades^2^. Gram-negative organisms in particular are estimated to account for between 26–47% of BSI in the United States and constitute an even higher proportion of cases in other parts of the world^3,4^. Furthermore, the prevalence of drug-resistance in organisms causing bacteremia is of major concern, as exemplified by a report that classified 33% of Gram-negative bacteria recovered from BSI as multi-drug resistant^5^. In addition to the highly prevalent species *Escherichia coli* and *Klebsiella pneumoniae*, there is a relatively small cohort of Gram-negative opportunistic pathogens that are also frequently associated with BSI, with *Citrobacter freundii* among these species^3,5–7^.

The genus *Citrobacter* in the *Enterobacteriaceae* family consists of 13 currently recognized species, and among these, *Citrobacter freundii* and *Citrobacter koseri* are the most frequently associated with infection of humans. *C. freundii*, the focus of this study, can be found in the intestinal tracts of humans and other animals and from diverse environmental sources^8–10^. Previous studies have described the isolation of enterotoxin-producing *C. freundii* in association with diarrhea^11–13^; however, such toxigenic *C. freundii* appear to be uncommon and the primary impact of *C. freundii* on human health is arguably as an opportunistic pathogen. *C. freundii* causes a broad spectrum of infections as an opportunistic pathogen, including infections of the urinary tract, respiratory tract, wounds, and bloodstream. Limited outbreaks involving clonal drug-resistant isolates have also been observed in healthcare settings^14–16^. In surveys from multiple North American healthcare facilities, 3–6% of *Enterobacteriaceae* infections were attributed to the *Citrobacter* genus^17,18^. As with other *Enterobacteriaceae* species, resistance to β-lactam antibiotics via the production of carbapenemases and the AmpC β-lactamase have also been documented and may complicate treatment of these infections^7,18–21^.

The primary pathogenesis of sepsis is the result of a dysregulated immune response, often originating from infection, that can be followed by organ failure and death in severe cases. It is well-established that pathogen-associated molecular patterns such as LPS, flagella, and peptidoglycan play a significant role in initiating the sepsis inflammatory immune response through pattern recognition receptors^22^ and it is also known that sepsis in model organisms can be recapitulated by exposure to these bacterial products. Together, these characteristics imply that persistence of bacteria in the bloodstream environment alone constitutes a primary driver of sepsis and that initiation of sepsis may be less dependent on canonical virulence strategies compared to other bacterial-mediated diseases. With this in mind, we sought to define the physiologic requirements for survival and fitness of the opportunistic pathogen *C. freundii* in a murine bacteremia model. The results of this work establish that fitness in this model system is largely dependent on fundamental bacterial processes, of which metabolism, protein secretion, and maintenance of genetic material are explored in more detail. The results with *C. freundii* are also analyzed in the context of our previous work using *Serratia marcescens* and a similar bacteremia model to identify common pathways of fitness in the bloodstream environment.

## Results

### Collection of Citrobacter BSI isolates

**Figure 1:**
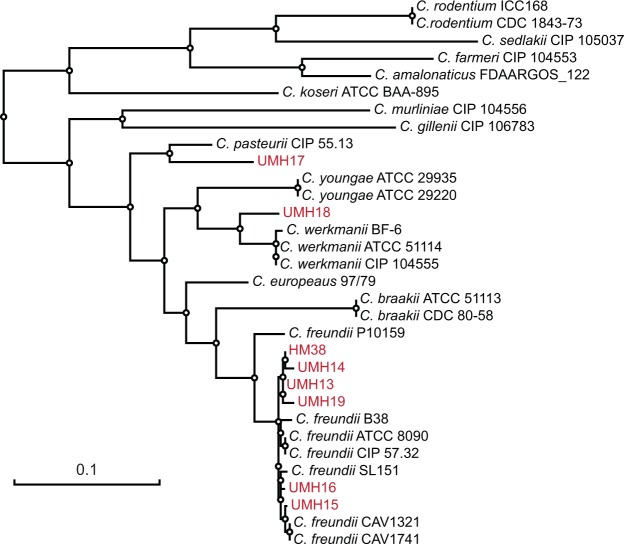
Phylogenetic distribution of strains collected in this study. The maximum-likelihood tree was generated from nucleotide sequences of concatenated *fusA*, *leuS*, *rpoB*, and *pyrG* alleles. Information from type strains was included where available and the strains collected in this study (UMH13–19 and HM38) are indicated by red font. The tree was manually rooted on the node between *C. koserii* and the eight species belonging to the *C. freundii* complex. The scale bar represents nucleotide substitutions per site.

Compared to other Gram-negative species that regularly cause BSI, limited information is available regarding the physiology of *C. freundii* within the host environment. With the goal of addressing this shortcoming, we first collected eight isolates belonging to the *C. freundii* complex from patients with BSI within the University of Michigan Health System. Ribosomal RNA-based phylogeny is capable of distinguishing three groups of *Citrobacter* species, of which, group I encompasses at least eight species and includes *C*. *freundii*^23,24^. However, 16S rRNA sequence and mass spectrometry-based identification approaches offer limited phylogenetic resolution within this group of *Citrobacter* species. Therefore, the isolates used in this study were further assessed using a multilocus sequence analysis approach. Among the eight collected isolates, six clustered closely with established *C. freundii* strains (Fig. 1) and had an average nucleotide identity of >98% with the type strain ATCC 8090, which is above the typically accepted 95% cutoff for isolates of the same species. Of the two remaining isolates, UMH17 clustered most closely with the *Citrobacter pasteurii* type strain CIP 55.13 and UMH18 with *Citrobacter werkmanii* strains.

### *C. freundii* bacteremia

Prior to investigating the fitness requirements of *Citrobacter* during BSI, a murine model of bacteremia was developed based on earlier work with other enteric species^25^. Among the *Citrobacter* BSI isolates, strains UMH14 and UMH15 were chosen as candidates for use in this study. Isolate UMH14 exhibited more consistent dose-dependent colonization of the spleen and liver at 24 hours following tail vein injection (Fig. S1) and was selected for further characterization. It was also necessary to test the infection model for potential colonization bottlenecks prior to assessing the contribution of individual *C. freundii* genes to fitness using a large collection of transposon mutants. A spontaneous nalidixic acid-resistant mutant of UMH14 (UMH14^Nal^) was isolated and determined to have equivalent *in vitro* fitness during co-culture with the parent strain in rich medium (Fig. S2). To determine if a diverse population of mutants could be established in the bloodstream without stochastic loss of individual clones, the UMH14^Nal^ and UMH14 strains were mixed at different ratios and inoculated into mice. After 24 hours, ratios of up to 1:10,000 (UMH14^Nal^:UMH14) were tolerated without spontaneous loss of the under-represented strain in the spleen (Fig. S2), indicating that a single mouse could accommodate at least 10,000 unique transposon mutants at a total dose of 5 × 10^7^ bacteria using this model.

**Figure 2:**
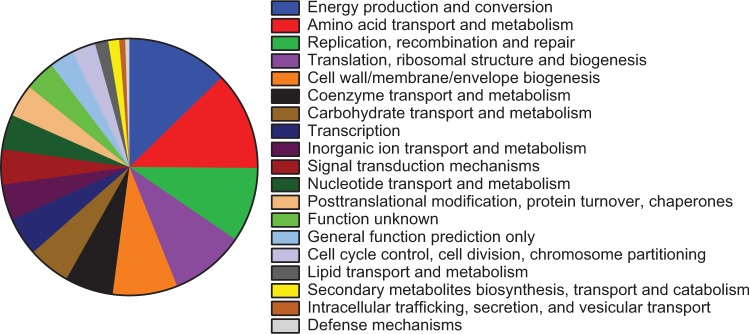
Distribution of *C. freundii* fitness factors by COG classification. Amino acid sequences of bacteremia fitness factors meeting the significance criteria were analyzed for COG distribution. COG classes not shown did not contain representative proteins among the 177 fitness factors. Twenty-two fitness factors were not categorized by this method.

To identify *Citrobacter* fitness genes in the bacteremia model, five pools of random transposon insertion mutants representing >44,000 unique insertion sites were injected into mice and bacteria colonizing the spleen were recovered after 24 hours (Fig. S3). The relative abundance of individual transposon mutants in the inoculum and splenic outputs, as determined by high-throughput sequencing, was used to identify genes that contribute to bacterial survival in the model. A total of 177 transposon-disrupted genes conferred a significant loss of fitness and nine genes were associated with increased bacterial fitness when mutated (fold-change ≥2.0, Adj. P < 0.05) (Data S1). A second analysis with this data set identified 546 chromosomally-encoded putative essential genes for which the number of reads in the input population was significantly lower than expected based on available TA sites and the library population size (logFC <−5.17) (Data S2). Using this model of opportunistic and systemic infection, it was anticipated that the majority of identified fitness factors would represent core physiologic processes rather than prototypical virulence factors (*e.g*., protein toxins or adhesins). Indeed, categorization of the 177 genes associated with significant fitness defects by Clusters of Orthologous Groups (COG) reflected the predominance of metabolic pathways and cellular maintenance functions required during *C. freundii* bacteremia (Fig. 2). Approximately half of the identified fitness genes fit broadly within five COG categories encompassing energy production, amino acid metabolism, DNA replication and repair, translation, and cell wall and membrane biogenesis.

### Validation of individual fitness gene mutants

**Figure 3:**
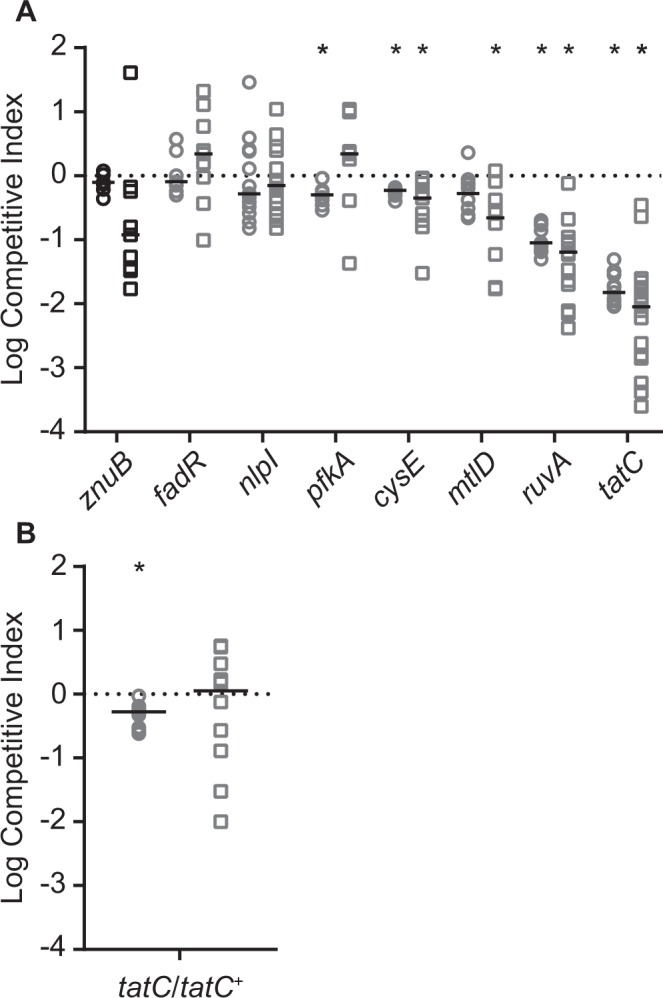
Competition infections with selected *C. freundii* mutants. (**A**) Mixtures (1:1) of the *C. freundii* UMH14 parent strain and mutant derivatives were co-inoculated into mice via tail vein injection. The number of wild-type and mutant bacteria present after 24 hours was used to calculate the CI for bacteria inhabiting the spleen (circles) and liver (squares). The *znuB* mutant was not anticipated to have a fitness defect and is differentiated by black symbols. A hypothetical CI of 1.0 indicating no change in relative fitness is represented by the dotted line. Asterisks indicate mutants that exhibited a significant decrease in median fitness (solid horizontal lines) compared to the hypothesized value as determined by Wilcoxon signed rank test (n ≥ 7, P < 0.05). (**B**) Relative fitness between wild-type strain UMH14 and the complemented *tatC* mutant (*tatC*/*tatC*^+^). Experiments and statistical analyses were conducted as described for panel A.

The contribution of individual gene products to *C. freundii* fitness was confirmed with independent deletion-insertion mutations constructed in selected UMH14 genes (Table 1). All engineered strains reported here lacked gross replication defects as determined by growth in rich medium (Fig. S4). For each gene listed in Table 1, fitness was measured by co-infecting individual mutants with the UMH14 parent strain. Five of the seven initially-tested mutants exhibited a statistically significant competitive disadvantage in either the spleen or liver (Fig. 3A), thereby affirming the results of the transposon screen. The *znuB* mutant was selected as a negative control for validation here since the results of the transposon screen indicated that no fitness defect was associated with this gene, despite evidence from other bacterial species demonstrating the contribution of the ZnuABC zinc transport system to murine infection^26–28^. In competition with UMH14, the *znuB* mutant failed to exhibit a significant fitness defect, in agreement with the expected results. The adjacent *znuA* (fold-change = 1.5, Adj. P = 0.527) and *znuC* (fold-change = 2.0, Adj. P = 0.035) genes of UMH14 also conferred either no fitness defect or a minimal defect when mutated by transposon insertion (Data S1).

**Table 1:** Fitness scores for selected *C. freundii* genes.

Gene	Locus_tag	Predicted product	Fitness defect^a^ FC (Adj. P)
*ruvA*	CUC46_10495	Holliday junction processing protein	39.1 (8.4 × 10^−5^)
*cysE*	CUC46_19530	L-serine-*O*-acetyltransferase	10.3 (6.6 × 10^−4^)
*nlpI*	CUC46_17375	lipoprotein	8.5 (2.7 × 10^−5^)
*tatC*	CUC46_20805	twin-arginine translocase	5.5 (<1.0 × 10^−10^)
*mtlD*	CUC46_19485	mannitol-1-phosphate 5-dehydrogenase	4.8 (<1.0 × 10^−10^)
*pepP*	CUC46_16060	Xaa-Pro aminopeptidase	4.5 (3.5 × 10^−3^)
*fadR*	CUC46_10120	transcriptional regulator of fatty acid metabolism	3.9 (2.3 × 10^−7^)
*sufI*	CUC46_16565	cell division protein	3.3 (1.2 × 10^−7^)
*pfkA*	CUC46_21195	6-phosphofructokinase	2.0 (3.2 × 10^−2^)
*znuB*	CUC46_10485	zinc ABC transporter permease	1.1 (9.0 × 10^−1^)

^a^Decrease in fitness as determined by the abundance of transposon mutants recovered from murine spleens relative to inputs as fold-change (FC) with adjusted P values (Adj. P).

Mutation of the *tatC* gene, encoding a component of the twin-arginine translocation system, resulted the greatest loss of fitness among all the mutants tested (Fig. 3). To determine whether the decreased fitness of this mutant could be restored through genetic complementation, plasmid pBBR1MCS-5 harboring the *tatC* open reading frame was introduced into the *tatC* mutant and the relative fitness of the resulting strain was tested. The *tatC* mutant initially exhibited a 67-fold fitness defect in the spleen in competition with the wild-type strain (Fig. 3A), whereas the complemented *tatC* mutant exhibited only a two-fold fitness defect under the same conditions (Fig. 3B). Similarly, the complemented *tatC* mutant was not significantly out-competed in the liver compared to UMH14. The pBBR1MCS-5 parent plasmid is known to be stably maintained during infection in the absence of selection^29^ and *in vitro* culture of the *C. freundii tatC* mutant harboring either pBBR1MCS-5 or the *tatC*^+^ complementation plasmid demonstrated no appreciable loss of plasmid over 24 hours in the absence of selection (Fig. S5). Together, these results firmly establish the requirement for TatC function during *Citrobacter* bacteremia.

### Conservation of fitness genes among *Citrobacter* isolates

**Figure 4:**
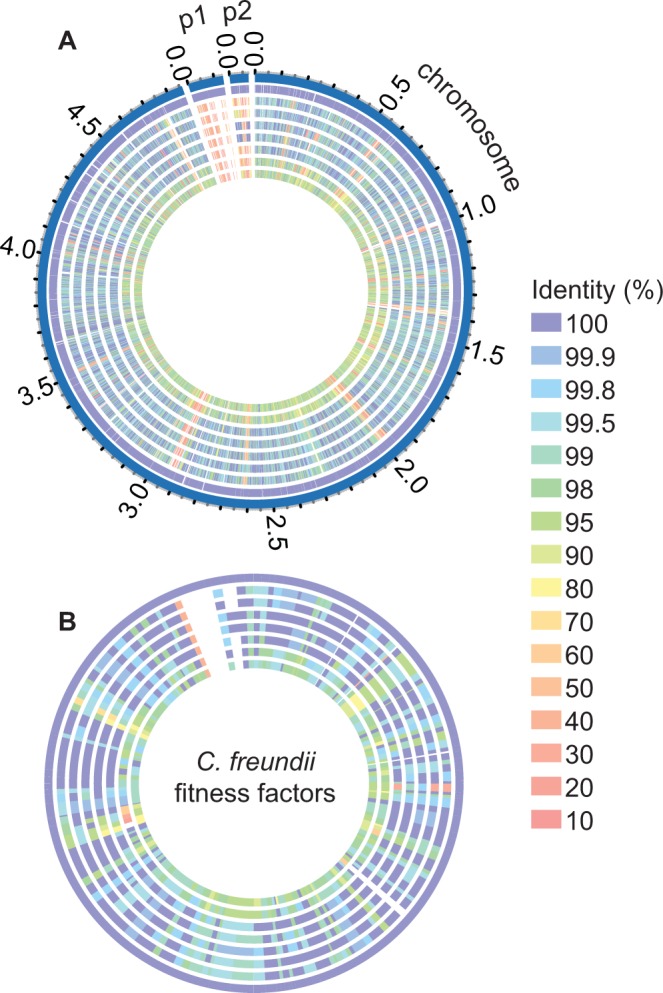
Proteome comparison of eight *Citrobacter* bacteremia isolates. (**A**) Predicted protein sequences from all *Citrobacter* isolates collected in this study were compared to the chromosome- and plasmid-encoded (p1 and p2) sequences of the reference strain UMH14 using the PATRIC webserver. (**B**) Amino acid sequences of the 177 *C. freundii* fitness factors were used as a reference to identify homologs within the other *Citrobacter* isolates. The level of amino acid sequence identity is indicated by color. Tracks from outside to inside: 1, UMH14; 2, UMH13; 3, UMH15; 4, UMH16; 5, UMH19; 6, HM38; 7, UMH17; 8, UMH18.

During the course of this study, the complete genome sequence of each *Citrobacter* isolate was determined and the predicted proteome of each strain was compared against UMH14 as a reference. Of the 4,666 predicted gene products encoded on the UMH14 chromosome, 3,742 are conserved at ≥95% identity between all *C. freundii* isolates in this study (Fig. 4A). The number of conserved proteins (≥95% identity) between all strains decreases to 2,545 when the predicted proteomes of UMH17 and UMH18 were included, consistent with the placement of these isolates outside the *C. freundii* branch by multilocus sequence analysis (Fig. 1). Conservation of UMH14 fitness factors was also high, with 145 of the predicted 177 proteins conserved at ≥95% amino acid identity among the eight bacteremia strains (Fig. 4B), including all seven of the initial fitness factors that were selected for validation (Table 1 and Fig. 3). Removing UMH17 and UMH18 from consideration increased the number of conserved fitness factors to 162 (92%). These results suggest a *Citrobacter* survival strategy during bacteremia that is largely dependent on conserved proteins within the species. Interestingly, few fitness factors were completely unique (<30% identity) to UMH14 within this subset of strains. One notable example is a putative three-gene operon (CUC46_23440-CUC46_23450) with observed fitness defects ranging between 6- and 14-fold. All three open reading frames encode hypothetical proteins and among these only CUC46_23450 is predicted to encode a conserved domain with an assigned function (cd01713, phosphoadenosine phosphosulfate reductase).

### DNA recombination and repair pathways

One of the goals of this study was to identify conserved biological pathways that involved multiple bacteremia fitness factors. The RecBCD and RuvABC enzyme complexes that are involved in DNA recombination and repair represent such an example. The RecBCD enzyme is active on duplex DNA ends and is required for the processes of homologous recombination and recombinational DNA repair. Related to these functions, the RuvABC enzyme facilitates Holliday junction branch migration and resolution^30,31^. Transposon insertion in either *recB*, *recC*, or *recD* resulted in a 6–8-fold reduction in fitness and similarly, disruption of both *ruvA* and *ruvC* by transposon insertion resulted in a >30-fold loss of fitness (Data S1). Importantly, the *ruvA* fitness defect was confirmed by competition infection against the wild-type strain using an independently-constructed mutant (Fig. 3A). Within the two multi-protein complexes, only RuvB was not identified as a significant fitness factor in our dataset. Both protein complexes are known to participate in the resolution of stalled or collapsed DNA replication forks that occur during normal chromosomal synthesis^30^, though importantly, the *ruvA* mutant grew similarly to the wild-type strain during rapid replication in rich medium (Fig. S4). It is tempting to speculate that bacterial DNA damage occurring in the host environment, potentially through immune cell-mediated production of reactive oxygen species, may also contribute to the requirement for these complexes.

### The twin-arginine translocation system

**Figure 5:**
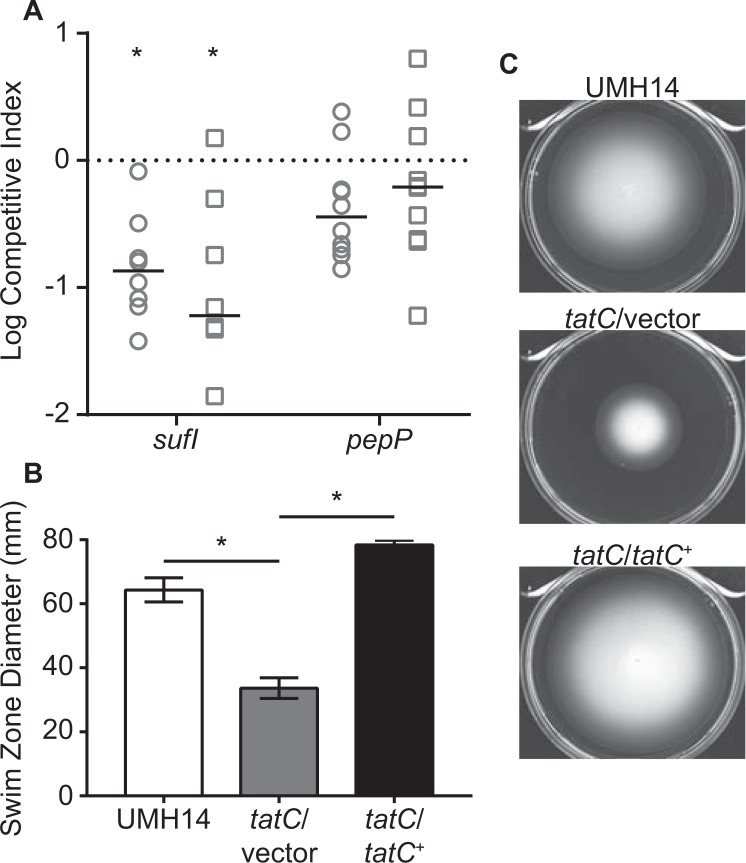
Fitness defects of putative Tat-secreted protein mutants and involvement of the Tat system in *C. freundii* motility. (**A**) Mixtures (1:1) of the *C. freundii* UMH14 parent strain and *sufI* or *pepP* mutants were used to co-inoculate mice via tail vein injection. The number of wild-type and mutant bacteria present after 24 hours was used to calculate the CI for bacteria inhabiting the spleen (circles) and liver (squares). A hypothetical CI of 1.0 indicating no change in relative fitness is represented by the dotted line. Asterisks indicate mutants that exhibited a statistically significant decrease in median fitness (solid horizontal lines) compared to the hypothesized value as determined by Wilcoxon signed rank test (n ≥ 8, P < 0.05). (**B**) Quantitation of swimming motility for wild-type, *tatC* mutant, and the complemented *tatC* mutant (*tatC*/*tatC*^+^) strains. Bacteria were inoculated into LB medium solidified with 0.3% agar and swimming motility was quantitated by measuring the diameter of the growth zone after incubation at 37 °C for 16 hours. Bars represent the mean from triplicate plates ± standard deviation. The *tatC* mutant exhibited significantly less motility than the wild-type and complemented mutant strains by *t*-test (asterisks, P < 0.001). (**C**) Representative agar plates demonstrating swimming motility.

The twin-arginine translocation system functions in Gram-negative bacterial species to secrete folded proteins across the cytoplasmic membrane. The role of TatC in this conserved multi-protein system is in the recognition of secretion signal sequences for proteins that are exported through this pathway. Having established the significant impact of the *tatC* gene on *in vivo* fitness of *C. freundii* (Fig. 3), it was reasoned that proteins secreted by the Tat system may also be required for fitness in the murine model. To identify putative Tat-secreted proteins, the N-terminal sequence of each of the 177 fitness factors was analyzed using the TatP 1.0 prediction software^32^. Two genes, CUC46_16565 and CUC46_16060, were identified as encoding Tat-dependent signal peptides containing twin-arginine motifs. The CUC46_16565 product shares 94% amino acid identity with the *E. coli* SufI protein, including 100% conservation of the twin-arginine motif and hydrophobic portion of the signal peptide^33^. The *C. freundii sufI* gene was mutated and fitness was assessed in competition with the UMH14 parent strain to determine if any of the fitness defect associated with the loss of TatC could be attributed to disruption of SufI function (Fig. 5A). Indeed, the *sufI* mutant strain was outcompeted by 7-fold in the spleen and 17-fold in the liver compared to the wild-type strain. However, since the fitness cost of the *tatC* mutation ranged from 67-fold to 112-fold in competition with wild-type bacteria (Fig. 3), the comparatively low fitness cost of the *sufI* mutation implied that additional Tat-dependent substrates may also contribute to fitness. Attempts to establish Tat-dependent secretion of SufI in *C. freundii* were unsuccessful due to apparent toxicity issues associated with the expression of a C-terminal FLAG epitiope-tagged SufI construct, specifically within the *tatC* mutant strain (data not shown). However, SufI has been used as a model Tat-secreted protein in numerous studies characterizing the *E. coli* Tat system.

The second potential Tat-secreted fitness factor that was identified, encoded by CUC496_16060, is a predicted proline aminopeptidase (PRK10879) belonging to the PepP superfamily. A mutant strain lacking the *pepP* gene was outcompeted by *ca*. 3-fold in the spleen and *ca*. 2-fold in the liver but the observed fitness disadvantage compared to wild-type was not statistically significant (Fig. 5A). Nonetheless, the subcellular localization of PepP was determined using a C-terminal FLAG epitope-tagged derivative carried on a multi-copy plasmid (PepP^FLAG^). Unexpectedly low levels of PepP^FLAG^ were detected in the *tatC* mutant compared to UMH14; however, the PepP^FLAG^ fusion protein was found primarily in the cytoplasmic fraction of both tested strains (Fig. S6) and no evidence for Tat-dependent subcellular localization of the PepP protein was obtained. Together with the *sufI* mutant competition infection results, these data further support the notion that additional Tat-dependent fitness factors are present in *C. freundii* or that the cumulative loss of protein translocation via the Tat system outweighs the loss of function for any single substrate.

One of the prevailing phenotypes associated with loss-of-function *tat* mutants across species is an impairment in motility^34–37^. *C. freundii* is a flagellated bacterium capable of swimming motility in a soft agar matrix. The *C. freundii tatC* mutant exhibited an approximately 2-fold reduction in swimming motility compared to the wild-type strain and swimming motility was fully restored by genetic complementation *in trans* (Fig. 5B,C). These results clearly demonstrate a motility defect in the absence of TatC function; however, since low-level swimming was still observed in the *tatC* mutant, some flagellar function is likely retained. With the exception of *fliQ*, the general lack of flagella-associated fitness genes identified during bacteremia suggests that the importance of *tatC* for *C. freundii* fitness may be largely independent of the flagella dysfunction observed in this strain.

### Metabolic fitness pathways

**Figure 6:**
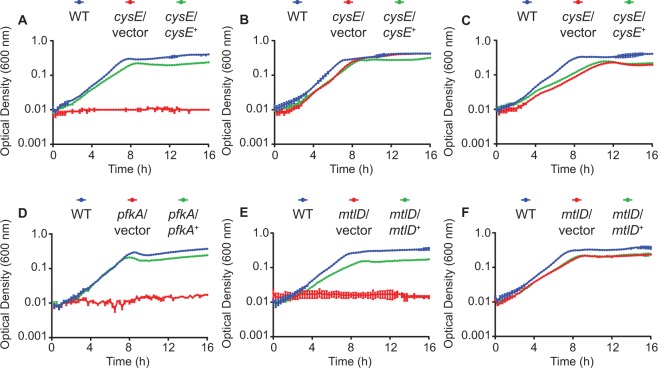
Growth of *C. freundii* metabolic mutants in defined medium. Wild-type (WT) *C. freundii* strain UMH14, mutants harboring vector control plasmids, and complemented mutants were cultured in defined M9 medium. Bacterial growth was measured by optical density (600 nm) in 15-minute intervals with each point representing the mean from triplicate cultures ± standard deviation. Supplements were added to the base M9 salts solution as follows: (**A**) 22 mM glucose; (**B**) 22 mM glucose and 10 mM OAS; (**C**) 22 mM glucose and 1 mM cysteine; (**D**) 22 mM glucose; (**E**) 22 mM mannitol; (**F**) 22 mM glucose.

As noted previously, a sizable portion of the identified *Citrobacter* fitness genes were predicted to function in metabolic pathways (Fig. 2). Three different genes representing components of central carbon metabolism (*pfkA*), fructose and mannose metabolism (*mtlD*), and amino acid biosynthesis (*cysE*) were chosen for further investigation. Bacterial cysteine biosynthesis occurs from L-serine through the intermediate *O*-acetyl-L-serine (OAS). This first step in the model *E. coli* pathway is catalyzed by the CysE *O*-acetyltransferase enzyme and the loss of *cysE* results in a cysteine auxotrophy^38,39^. A *C. freundii cysE* mutant is likewise unable to replicate in defined medium lacking cysteine (Fig. 6A) but can achieve near-wild-type densities when the medium was supplemented with either OAS (Fig. 6B) or cysteine (Fig. 6C). Genetic complementation of the *cysE* mutation resulted in partial restoration of growth in the absence of cysteine. Interestingly, the complete lack of growth for *cysE* mutant bacteria in the absence of OAS or cysteine *in vitro* is in contrast to the partial fitness defect observed during infection (Fig. 3A). This suggests that *Citrobacter* may be able to obtain these metabolites from the bloodstream environment, yet there is still a fitness cost associated with disruption of the biosynthetic pathway.

As part of our investigation into the host-associated metabolism of *Citrobacter*, the ability of this bacterium to utilize different carbon sources was investigated. The PfkA phosphofructokinase enzyme of other enteric species has been extensively characterized and represents the first committed step in glycolysis, catalyzing the phosphorylation of fructose-6-phosphate into fructose-1,6-bisphosphate. For UMH14, mutation of *pfkA* eliminated the ability of this strain to replicate on glucose as a sole carbon source (Fig. 6D), which could be partially restored by providing *pfkA* on a multicopy plasmid. This total loss of glucose utilization *in vitro* correlated to a two-fold decrease in fitness during bacteremia (Fig. 3A). As glucose is an abundant carbon source in mammalian serum^40^, these results are consistent with a role for *Citrobacter* utilization of glucose during infection, but also suggest that the metabolic repertoire of *Citrobacter* can facilitate the utilization of alternative carbon and energy sources.

A second enzymatic activity involving fructose-6-phosphate was also investigated for contributions to *Citrobacter* bacteremia. The mannitol-1-phosphate-5-dehydrogenase protein, MtlD, facilitates bidirectional conversion of mannitol-1-phosphate and fructose-6-phosphate using NAD^+^ or NADH as a co-factor^41,42^. Multiple enteric bacterial species can utilize the sugar alcohol mannitol as a sole carbon source following transport via a hexitol phosphoenolpyruvate-dependent phosphotransferase system, resulting in the intracellular accumulation of mannitol-1-phosphate^43,44^. One possible fate of mannitol-1-phosphate is MtlD-dependent oxidation to fructose-6-phosphate and subsequent funneling into the glycolytic pathway. The *C. freundii mtlD* mutant exhibited a five-fold decrease in fitness in the murine liver (Fig. 3A) and this observation prompted experiments to determine if *C. freundii* was capable of this type of metabolism *in vitro*. UMH14 and *mtlD* derivative strains were cultured in defined medium containing mannitol and the resulting growth curves demonstrate that wild-type bacteria and the complemented mutant were able to proliferate under these conditions while the *mtlD* strain could not (Fig. 6E). As expected, the *mtlD* mutant was able to replicate to near wild-type levels when provided with glucose as a carbon source under aerobic conditions (Fig. 6F). Together these results establish both the ability of *C. freundii* to utilize mannitol as a sole carbon source and the requirement for *mtlD* in this process.

### Shared fitness strategies between pathogens in the bloodstream environment

We have previously assessed the fitness requirements of another opportunistic pathogen, *S. marcescens*, using a similar murine model of bacteremia. The results of the *S. marcescens* study included the identification of 212 fitness genes using techniques similar to the present work^45^. In an effort to determine if these species share any common pathways for fitness during BSI, the predicted proteomes of both species were first compared. Proteins were considered homologs between *C. freundii* and *S. marcescens* if they shared ≥70% amino acid identity over ≥50% of the sequence. In total, 42 homologous proteins were identified as significant fitness factors in both organisms (Table S1) with a number of diverse functions are represented in this list of shared fitness factors. Notably, the RuvA protein, the loss of which resulted in a >10-fold decrease in *C. freundii* fitness by competition infection (Fig. 3A), was identified together with RuvC as fitness factors in *S. marcescens*. These results further support the hypothesis that resolution of Holliday junction complexes, potentially during recombinational repair of DNA damage, is important for *in vivo* fitness of these organisms. The PfkA phosphofructokinase enzyme was also identified as a fitness factor in both species. As observed with *Citrobacter, S. marcescens pfkA* is required for utilization of glucose as a sole carbon source, and was further required for optimal bacterial replication in heat-inactivated human serum^45^. In both organisms, *pfkA* contributed to fitness in the spleen as assessed by the transposon screen, though interestingly, the *S. marcescens pfkA* mutant also exhibited *ca*. 9-fold reduced fitness in the kidney by competition infection with the wild-type strain. In the course of this work, it was observed that *C. freundii* UMH14 wild-type strain exhibited relatively poor colonization of the kidney in the BSI model, but the *pfkA* gene of *Proteus mirabilis* has been shown to contribute to fitness in the kidney following urinary tract infection^46^. Together these results demonstrate the feasibility of identifying conserved fitness strategies across organisms within a specific environment. Further work will be needed to determine if these gene products are also required in additional BSI-causing organisms and if these conserved fitness pathways can be exploited.

## Discussion

Interactions between *C. freundii* and healthy individuals is generally considered to be non-pathogenic in nature; however, upon colonization of the bloodstream *C. freundii* is capable of causing a life-threatening infection leading to sepsis. As such, *C. freundii* belongs to a relatively small group of opportunistic Gram-negative bacterial species that are encountered with frequency in healthcare settings and are capable of causing a variety of infections in individuals with diverse underlying conditions^3,5^. The present study represents the first global approach to identifying the genetic requirements of *C. freundii* fitness in a mammalian bacteremia model. As a result, we have identified 177 individual fitness genes in this model and independently confirmed the required contribution of multiple metabolic pathways, Tat-dependent protein secretion, and DNA recombination and repair functions for *C. freundii* survival in the bloodstream. All five fitness factors that were confirmed through analysis of independent mutants were conserved at >95% amino acid identity among the eight sequenced *Citrobacter* isolates in this study and >80% of the total fitness factors identified in *C. freundii* UMH14 were conserved among the same eight strains, despite evidence that two of these isolates belong to different species of *Citrobacter*. Furthermore, we have begun to identify common pathways for fitness between members of this healthcare-associated class of opportunistic pathogens by comparing our previous results with *S. marcescens*. The identification of multiple homologous fitness factors between these two species suggests that bacterial survival and proliferation in the bloodstream depends on conserved processes. With further characterization of these common fitness strategies and expansion to other related bacterial pathogens, it is conceivable that novel strategies targeting conserved fitness pathways in these bacteria could be developed for therapeutic intervention. It is important to note that we have also established clear evidence for differential fitness requirements between related bacterial organisms within the same environment. While the list of gene products that were only identified in one of the two organisms is extensive, a set of fitness determinants that exemplifies this type of difference between *C. freundii* and *S. marcescens* is the latter’s production of a polysaccharide capsule. Eleven open reading frames within the 18-gene capsule locus of *S. marcescens* were shown to contribute to bacteremia in our previous work and the capsule was additionally demonstrated to be important for serum resistance of this organism^45^. Some of the core components of *S. marcescens* capsular polysaccharide transport, encoded by the *wza*, *wzb*, and *wzc* genes, are conserved in *C. freundii* but are located with a putative colonic acid biosynthetic gene cluster and were not found to be fitness genes for *C. freundii* during bacteremia. Likewise, additional *Serratia* capsule transport and assembly fitness genes, including *wzx* and *wzy*, could not be identified in UMH14.

In the predominant *E. coli* model of Tat secretion, TatB and TatC form a complex in the cytoplasmic membrane that act as a receptor for recognition of Tat-dependent signal peptides. Folded proteins are then translocated across the inner membrane via oligomeric TatA in a process dependent on the proton motive force^47^. Of the *C. freundii* fitness genes validated by competition infections, the *tatC* mutant strain exhibited the greatest defect. The contribution of the Tat secretion complex to bacterial survival during mammalian infection has previously been demonstrated, but only for a subset of species in which this widely-conserved secretion system is present. In the case of *Pseudomonas aeruginosa*, disruption of the Tat system results in reduced rat lung abscess formation^35^ and reduced secretion of virulence-associated phospholipase C enzymes^48^. *Salmonella enterica* serovar Typhimurium *tatC* mutants exhibit colonization defects in the liver and spleen following intraperitoneal inoculation of mice^49^. A second study attributed the majority of *Salmonella tat* mutant attenuation to the simultaneous mis-localization of three proteins, AmiA, AmiC, and SufI^50^, though in this same work, the *Salmonella sufI* single mutant did not exhibit any colonization defect. *Yersinia pseudotuberculosis tatC* mutants are also attenuated following oral inoculation of mice^37^ and in this case, a *sufI* mutation alone recapitulates the full attenuation level observed for the *tatC* mutant^51^. Our results indicate that the SufI protein of *C. freundii* is likely Tat-secreted based on the presence of a conserved signal sequence and we have demonstrated that mutation of *C. freundii sufI* results in a 7- and 17-fold fitness defect in the spleen and liver, respectively (Fig. 5). Compared to the fitness defect of the *C. freundii tatC* mutant (67–112 fold), it is reasonable to conclude that Tat-dependent functions beyond the predicted translocation of SufI contribute to the loss of fitness in the *tatC* mutant. Although we were unable to identify additional Tat substrates associated with fitness in the transposon screen, experimental determination of the Tat-dependent proteome in *C. freundii* could reveal additional fitness factors. Mutations in genes encoding Tat components are known to confer pleotropic phenotypes including defects in anaerobic respiration, motility, metal homeostasis, acid and detergent resistance, and membrane integrity^35,52–55^. Therefore, it is possible that similar phenotypes may also contribute to the fitness defect of the *C. freundii tatC* mutant. The molecular basis for the requirement of SufI in the bloodstream environment remains to be determined. This protein is thought to provide cell division functions based in part on co-localization with the septal ring^56^ and genetic analyses demonstrating suppression of divisome mutants via overexpression of SufI^57,58^. However, the reduced fitness of *sufI* mutants during infection is intriguing, since we and others have observed that SufI is not essential for bacterial cell division in standard laboratory media (data not shown)^50,51^.

Between our previous study^45^ and the present work, we have begun to identify metabolic pathways that contribute to the fitness of opportunistic pathogens in the bloodstream environment. The ability to transport and utilize nutrients in this environment is critical for the establishment of infection and the enzyme-rich nature of these metabolic processes makes future disruptive strategies using small molecule inhibitors attractive. The observed fitness defect of the *Citrobacter mtlD* mutant is particularly intriguing given the relatively low levels of mannitol present in the mammalian bloodstream compared to other potential carbon sources^40^. Although the anticipated function of the *Citrobacter* MtlD protein was demonstrated genetically by forcing bacteria to utilize exogenous mannitol as a carbon source, a potential lack of MtlD-dependent reduction of fructose-6-phosphate into mannitol-1-phosphate should not be excluded as a cause for the observed *mtlD* mutant fitness defect in the host. Therefore, the possible role of MtlD in re-generation of the NAD^+^ pool via the reduction of fructose-6-phosphate during growth in the presence of blood-glucose should also be considered^59^. Similar to the observations that resulted from disruption of the glycolytic pathway, mutation of *mtlD* in *Citrobacter* also conferred a partial fitness defect. Together these results suggest either that the primary host carbon source remains to be identified or that *C. freundii* can compensate for the loss of individual carbon utilization pathways in the host environment. Additional work is needed to identify potential intersection points within the metabolic networks of this organism and others to identify targets that have maximal fitness effects.

*C. freundii* represents an understudied member of the *Enterobacteriaceae* family with relevance to healthcare-associated opportunistic infections. By defining the biological pathways for fitness in the mammalian bloodstream, the results reported herein emphasize the importance of fundamental *Citrobacter* physiology for replication and survival in the host environment and will provide a framework for further investigation into the nature of *Citrobacter* infections.

## Materials and Methods

### Bacterial strains and culture conditions

The *Citrobacter* isolates used in this study were obtained through the University of Michigan Clinical Microbiology Laboratory from patients with BSI. Bacterial isolates were obtained with approval from the University of Michigan Institutional Review Board and no identifying patient data was collected. All strains were identified as members of the *C. freundii* complex during routine clinical laboratory typing procedures. The *C. freundii* UMH14^Nal^ strain was obtained by selecting for spontaneous resistance to nalidixic acid on agar plates and engineered mutants of UMH14 are listed in Table S2. *Citrobacter* and *E. coli* strains were routinely cultured in LB medium^60^ unless indicated otherwise. Antibiotics were added to broth and agar medium at the following concentrations: ampicillin, 100 µg/ml; gentamicin, 10 µg/ml; hygromycin B, 200 µg/ml; kanamycin, 50 µg/ml; nalidixic acid, 50 µg/ml.

*C. freundii* mutant strains were assayed for growth defects in comparison with the parent strain UMH14 by culturing in LB medium from an initial optical density (600 nm, OD_600_) of 0.01. For growth analysis in defined conditions, strains were cultured overnight in LB then washed in PBS before subculture into M9 medium^61^. The M9 base medium was supplemented with 22 mM glucose, 22 mM mannitol, 1 mM L-cysteine, or 10 mM OAS as indicated. Growth curves were generated using a BioScreen C Analyzer set to 37 °C with continuous shaking and OD_600_ values were recorded in 15-minute intervals.

### Genome sequencing and phylogenetic analysis

Genomic DNA for sequencing was isolated from *Citrobacter* strains using Qiagen 100/G genomic tips according to the manufacturer’s recommendations. Whole-genome sequencing was performed at the University of Michigan DNA Sequencing Core Facility using the PacBio RS II platform and reads were assembled by the University of Michigan Bioinformatics Core Facility using the PacBio SMRT portal. All putative plasmids were verified via PCR and additional sequencing. The position of the first nucleotide in each genome was set based on the *E. coli* convention. Preliminary annotations, including the UMH14 annotation used for transposon mutant read-mapping, was performed via Rapid Annotation using Subsystem Technology (RAST)^62^. Sequences were annotated using the Prokaryotic Genome Annotation Pipeline^63^ upon NCBI deposition.

The multi-locus sequence analysis scheme^24,64^ used concatenated sequences internal to the *fusA*, *leuS*, *rpoB*, and *pyrG* open reading frames and was performed with PhyML 3.0^65^ and Smart Model Selection^66^. Type strains of each *Citrobacter* species were included where available. Further support for the species designations in this work was obtained by average nucleotide identity using the JSpeciesWS BLAST algorithm^67^.

### Predicted proteome analysis

Homologous protein sequences among the *Citrobacter* isolates were identified using the PATRIC proteome comparison tool^68,69^. For both the whole-genome comparison and the fitness factor comparison, amino acid sequence identity was limited to ≥10% over a minimum of 30% of the sequence. Plasmid sequences, where present, were included in the analysis. Predicted protein homologs between *S. marcescens* UMH9 and *C. freundii* UMH14 (≥70% sequence identity, ≥50% coverage) served as the basis for identification of shared fitness genes between the two species. Functional classification of fitness factors was accomplished using the webMGA server to perform RPSBLAST against the COG database^70^.

Putative Tat substrates were identified using the TatP 1.0 prediction software^32^. Analysis was limited to the first 100 amino acids of each of the 177 predicted UMH14 bacteremia fitness factors and used the default signature motif search function. Only proteins with appropriate signal peptide sequences and a twin-arginine signature motif were considered.

### Genetic manipulation of *C. freundii*

The *C. freundii* UMH14 mutant library was generated by transposon insertion following S17-1 mediated conjugative transfer^71^ of suicide plasmid pSAM_Cam (Bachman, 2015). Recipient bacteria were treated at 42 °C for 20 minutes prior to mixing 1:1 with the donor strain and conjugation was allowed to proceed for 6 hours. Transposon mutants were combined in pools of ~10,000 and stored at −80 °C prior to use for *in vivo* screening. Random insertion of transposons into the *C. freundii* UMH14 genome was determined by Southern blot. Genomic DNA isolated from putative mutants was fragmented by HindIII digestion and probed using a DIG-labeled PCR product that consisted of a 501-bp internal portion of the transposon-encoded kanamycin resistance gene^72^.

*C. freundii* UMH14 harboring the temperature-sensitive plasmid pSIM18^73^ was mutated by λ Red recombineering based on the methods of Thomason *et al*.^74^. Deletion-insertion alleles were generated by PCR using oligonucleotides having ~50 bases of target gene homology (Table S3) and the kanamycin resistance gene of pKD4^75^ as a template. DpnI-treated PCR products were introduced into the UMH14/pSIM18 strain via electroporation and putative recombinants were verified by PCR amplification and sequencing. All mutant strains generated in this study were cured of the pSIM18 recombineering plasmid prior to experimental analysis^74^.

### Molecular cloning and complementation

A 916-bp fragment containing the *tatC* gene from UMH14 was PCR amplified and ligated to pCR^TM^4Blunt-TOPO^®^ (ThermoFisher). The resulting plasmid was digested with EcoRI and HindIII and the *tatC*-containing fragment was ligated to pBBR1MCS-5^29^ to create the *tatC* complementation plasmid. Complementation of the *pfkA* mutation was accomplished by ligating a 1508-bp fragment containing the *pfkA* open reading frame with PCR-generated HindIII and XbaI restriction sites directly to appropriately digested pBBR1MCS-5. For complementation of the *mtlD* and the *cysE* mutations, fragments containing the *mtlD* (1,593 bp) and *cysE* (1,476 bp) open reading frames were first PCR amplified and ligated to the pCR™ 2.1-TOPO^®^ TA vector (ThermoFisher). Both insert fragments were subcloned via BamHI and PstI digestion and subsequent ligation to pBBR1MCS-5. The PepP^FLAG^ construct was generated by site-directed mutagenesis of the *pepP* gene harbored on plasmid pBBR1MCS-5. Oligonucleotide primers encoding the DYKDDDDK amino acid sequence were used to insert the FLAG epitope immediately upstream of the stop codon in the *pepP* open reading frame. Oligonucleotide primers used for the construction of complementation plasmids are listed in Table S3 and all recombinant plasmids were confirmed by sequencing.

The stability of pBBR1MCS-5 in *C. freundii* was determined in the absence of selection prior to complementation during infection. UMH14 harboring the empty vector or the *tatC*-containing plasmid was cultured overnight in LB medium with gentamicin. Bacteria were collected by centrifugation and washed twice with PBS to remove residual antibiotic. Washed bacteria were subcultured into LB at a low initial density and incubated at 37 °C. The proportion of bacteria harboring plasmids was determined after 24 hours by serial dilution and plating on LB agar with or without gentamicin.

### Murine model of *C. freundii* bacteremia

Bacteria for murine infection experiments were prepared by subculturing overnight LB growth into fresh medium and incubating for 2.5 hours. Exponential phase bacteria were collected by centrifugation and resuspended in an appropriate volume of PBS. Female 6–8 week old C57BL/6 mice were infected with *ca*. 5 × 10^7^ CFU via tail vein injection, unless otherwise noted. The spleen and liver from mice sacrificed at 24 hours post-inoculation were homogenized in PBS and ten-fold serial dilutions were plated on LB agar to determine the bacterial burden. For competition infections, wild-type *C. freundii* was mixed with antibiotic-resistant mutant strains at a 1:1 ratio prior to infection. The viable count for each strain was determined for both the inoculum (input) and organ homogenates (output) by serial dilution and differential plating on LB and LB containing antibiotics. The competitive index (CI) was calculated as follows: (CFU_mutant_/CFU_wild-type_)^output^/(CFU_mutant_/CFU_wild-type_)^input^. All murine infections were conducted using protocols approved by the University of Michigan Institutional Animal Care and Use Committee and in accordance with the Office of Laboratory Animal Welfare guidelines.

The relative fitness of strains UMH14^Nal^ and UMH14 was initially determined *in vitro* by co-culture in LB medium at ratios of 1:100 and 1:1,000 (UMH14^Nal^:UMH14). For bottleneck assessment, the two strains were cultured independently as described for the experimental infections and then mixed at 1:1, 1:1,000, 1:5,000, and 1:10,000 ratios (UMH14^Nal^:UMH14). Mice infected with these mixtures were sacrificed after 24 hours and the spleens were excised and homogenized in PBS. To ensure sufficient recovery of the UMH14^Nal^ strain in the output population at each of the tested ratios, it was necessary to expand the entire population by mixing 2 ml of spleen homogenate with 3 ml of LB and incubating for 5 hrs at 37 °C prior to determining the CI. Culture suspensions were serially diluted and differentially plated on LB agar and LB containing nalidixic acid for calculation of the CI. The CI from mice infected with the 1:1 ratio was determined both before and after outgrowth in LB medium as a control.

### Screen for *C. freundii* fitness genes during BSI

Five pools of ~10,000 mutants each were propagated for 2.5 hours in LB before collecting bacteria by centrifugation and resuspending to a density of *ca*. 5 × 10^8^ CFU/ml. Bacterial suspensions in PBS were used to infect four replicate mice for each of five mutant pools. Aliquots from the remaining bacterial suspensions were reserved for the preparation of genomic DNA to serve as input samples. Mice were sacrificed 24 hours after inoculation and spleens were homogenized in PBS. A small volume of each homogenate was used to determine the bacterial burden by serial dilution and plating and the remainder was plated on LB for recovery of output bacteria. One mouse failed to yield a productive infection and was eliminated from further analysis (pool 1, n = 3). Outputs from individual mice were maintained separately throughout the analysis and inputs were processed as technical duplicates. Genomic DNA^76^ from input and output bacterial samples was used as template for PCR amplification of transposon insertion site sequences as described previously^77^. Purified PCR products containing transposon-chromosome DNA junctions were sequenced on the Illumina HiSeq platform using 50-cycle single-end reads by the University of Michigan DNA Sequencing Core Facility.

Sequence reads were mapped to the UMH14 genome and essential genes that are non-permissive to transposon insertion were identified using the ESSENTIALS pipeline^78^. User-defined parameters for the ESSENTIALS analysis employed the default settings with the following exceptions: library size was set to 50,000, zero barcode mismatches were allowed, and the Cox-Reid method of dispersion estimation was used in edgeR. Two output samples failed to return sufficient mapped reads for inclusion in the data set and were eliminated from further analysis (pool 4, n = 3; pool 5, n = 3). Fitness genes were identified using the TnseqDiff function in the Tnseq R package^79^. The fitness genes reported herein were identified based on the statistical cutoff of fold-change >2.0 and adjusted *P* < 0.05.

### Motility assays

*C. freundii* swimming motility was assayed using LB medium solidified with 0.3% agar. Bacteria from single colonies were stab-inoculated into the center of the agar and incubated at 37 °C for 16 h. Motility was measured by the diameter of the resulting growth zones and representative plates were imaged using a Q-Count automated colony counter.

### Subcellular fractionation and immunoblotting

Subcellular fractions were prepared from both the wild-type and *tatC* mutant strain harboring the *pepP*^FLAG^ allele on plasmid pBBR1MCS-5. *C. freundii* strains cultured in LB medium were harvested in early exponential-phase by centrifugation at 11,000 × *g* for 10 minutes. The cell-free supernatant was concentrated by trichloroacetic acid precipitation in the presence of sodium deoxycholate^80^ and was used as the extracellular fraction. The bacterial pellet was resuspended in 5 ml of 0.5 M Tris-HCl (pH 7.8) and collected again by centrifugation, then resuspended in ice-cold osmotic shock buffer (0.2 M Tris-HCl pH 8.0, 1 M sucrose, 1 mM EDTA). Following centrifugation and removal of the supernatant, cells were quickly re-suspended in ice-cold water, resulting in the liberation of the periplasmic fraction. Following osmotic shock, cells were collected by centrifugation and re-suspended in 50 mM Tris-HCl (pH 7.8) before being ruptured by two passes through a French pressure cell (20,000 psi). The lysate was subjected to centrifugation at 200,000 × *g* for 30 minutes to separate the soluble cytoplasmic fraction and the crude membrane fraction. The membrane fraction was rehydrated in 50 mM Tris-HCl pH 7.8 prior to analysis. All subcellular protein fractions were separated by SDS-PAGE, followed by transfer to PVDF membrane and immunoblotting using standard techniques^81,82^. Total proteins in each fraction were normalized between strains by Coomassie staining prior to immunodetection experiments. The FLAG epitope was detected with a mouse anti-FLAG M2 monoclonal antibody (Sigma). Primary antibodies against *E. coli* MBP (New England BioLabs) and RpoB (BioLegend) were used as fractionation controls. A goat anti-mouse IgG HRP-conjugated secondary antibody (Kirkegaard and Perry Laboratories) was used for all blots.

### Data availability

Transposon insertion site sequences were deposited in the NCBI Sequence Read Archive (SRP119409). Genomic sequences of *Citrobacter* BSI isolates are available under Genbank accession numbers CP024672- CP024683.

## Electronic supplementary material

Supplementary Material

Dataset 1

Dataset 2
