# Supplementary material for: Citrobacter freundii fitness during bloodstream infection
Source: Sci Rep. 2018 Aug 7;8:11792. doi: 10.1038/s41598-018-30196-0 (PMC6081441; doi:10.1038/s41598-018-30196-0)
Supplement: Supplementary file 1 — Supplementary Material [file 41598_2018_30196_MOESM1_ESM.pdf]

Citrobacter freundii fitness during bloodstream infection

Mark T. Anderson, Lindsay A. Mitchell, Lili Zhao, and Harry L. T. Mobley

SUPPLEMENTAL TABLES

Table S1. Shared *S. marcescens* and *C. freundii* fitness factors.

Locus_tag			Fold fitness defect (Adj. P)	
S. marcescens	***C. freundii***	**Product (RAST)**	***S. marcescens***	***C. freundii***
BVG96_00190	CUC46_00255	SSU rRNA dimethyltransferase	66.4 (1.2E-04)	2.7 (3.3E-03)
BVG96_01810	CUC46_02125	Cytochrome O ubiquinol oxidase subunit I	20.7 (3.1E-07)	2.2 (3.3E-04)
BVG96_02425	CUC46_03225	Lipoate synthase	22.5 (2.4E-05)	4.4 (1.6E-02)
BVG96_02675	CUC46_03505	Negative modulator of initiation of replication, SeqA	297.0 (<1.0E-10)	7.2 (1.6E-04)
BVG96_05150	CUC46_04600	Thioredoxin reductase	19.4 (1.1E-02)	6.8 (1.6E-04)
BVG96_06525	CUC46_09690	GTP-binding and nucleic acid-binding protein, YchF	15.2 (<1.0E-10)	3.0 (1.2E-02)
BVG96_07075	CUC46_10340	Influences osmotic activation of compatible solute ProP, ProQ	62.6 (<1.0E-10)	19.6 (4.9E-10)
BVG96_07080	CUC46_10335	Tail-specific protease precursor	18.3 (5.5E-09)	9.0 (<1.0E-10)
BVG96_07525	CUC46_07550	Purine nucleotide synthesis repressor	30.1 (2.2E-05)	3.1 (1.5E-02)
BVG96_10320	CUC46_10160	Septum site-determining protein, MinD	174.2 (2.7E-05)	2.9 (6.6E-04)
BVG96_10425	CUC46_10495	Holliday junction DNA helicase, RuvA	24.6 (2.0E-07)	39.1 (8.4E-05)
BVG96_10430	CUC46_10500	Crossover junction endodeoxyribonuclease, RuvC	45.9 (3.9E-10)	31.3 (7.4E-03)
BVG96_13365	CUC46_12685	Acetate kinase	2.5 (2.6E-02)	13.1 (1.5E-07)
BVG96_13370	CUC46_12690	Phosphate acetyltransferase	3.2 (3.9E-03)	3.5 (5.7E-03)
BVG96_13495	CUC46_12805	tRNA pseudouridine synthase A	19.2 (3.0E-05)	2.2 (2.5E-02)
BVG96_15700	CUC46_00935	Protein-PII uridylyltransferase	14.5 (5.8E-04)	3.1 (2.4E-05)
BVG96_15885	CUC46_15710	Adenosine (5')-pentaphospho-(5")-adenosine pyrophosphohydrolase	70.0 (<1.0E-10)	3.2 (1.1E-02)
BVG96_16230	CUC46_15935	Tyrosine recombinase, XerD	64.3 (<1.0E-10)	3.8 (3.7E-05)
BVG96_16880	CUC46_00615	Dihydrolipoamide dehydrogenase	3.8 (5.8E-03)	23.0 (3.0E-04)
BVG96_16890	CUC46_00605	Pyruvate dehydrogenase E1 component	9.3 (<1.0E-10)	2.7 (5.1E-08)
BVG96_17650	CUC46_17625	Aerobic respiration control sensor, ArcB	9.1 (<1.0E-10)	10.3 (<1.0E-10)
BVG96_17690	CUC46_17695	Stringent starvation protein A	8.7 (2.2E-08)	13.6 (5.4E-08)
BVG96_18955	CUC46_18465	3-dehydroquinate synthase	18.6 (<1.0E-10)	8.3 (1.7E-09)

BVG96_19960	CUC46_21195	6-phosphofructokinase	56.9 (<1.0E-10)	2.0 (3.2E-02)
BVG96_20130	CUC46_19660	LSU ribosomal protein L33p	7.2 (2.8E-06)	99.1 (2.5E-02)
BVG96_20255	CUC46_20970	GTP-binding protein, TypA/BipA	57.1 (<1.0E-10)	3.4 (5.0E-09)
BVG96_20265	CUC46_20960	Glutamine synthetase type I	4.4 (1.5E-02)	2.7 (5.5E-05)
BVG96_20305	CUC46_20925	DNA polymerase I	37.8 (<1.0E-10)	3.9 (<1.0E-10)
BVG96_20435	CUC46_20345	rRNA small subunit 7-methylguanosine methyltransferase, GidB	17.9 (1.7E-03)	3.3 (8.4E-05)
BVG96_20445	CUC46_20335	ATP synthase F0 sector subunit A	9.2 (3.8E-02)	4.1 (1.2E-05)
BVG96_20465	CUC46_20315	ATP synthase alpha chain	19.8 (4.5E-03)	2.7 (2.3E-02)
BVG96_20470	CUC46_20310	ATP synthase gamma chain	23.2 (5.3E-09)	3.7 (8.2E-04)
BVG96_20475	CUC46_20305	ATP synthase beta chain	10.4 (1.3E-05)	3.2 (4.9E-06)
BVG96_21195	CUC46_20555	Undecaprenyl-phosphate N-acetylglucosaminyl 1-phosphate transferase	7.0 (<1.0E-10)	2.4 (1.6E-05)
BVG96_21235	CUC46_20595	WzxE	115.4 (<1.0E-10)	3.6 (2.4E-05)
BVG96_21325	CUC46_20685	Tyrosine recombinase, XerC	17.6 (1.5E-08)	20.5 (<1.0E-10)
BVG96_22100	CUC46_22565	Lysyl-lysine 2,3-aminomutase	7.5 (5.9E-05)	3.1 (3.7E-02)
BVG96_22150	CUC46_22630	Translation elongation factor P Lys34:lysine transferase	20.3 (5.2E-07)	2.8 (1.1E-02)
BVG96_22245	CUC46_22730	Adenylosuccinate synthetase	21.9 (<1.0E-10)	2.4 (3.6E-02)
BVG96_22550	CUC46_17380	Polyribonucleotide nucleotidyltransferase	37.1 (<1.0E-10)	5.9 (1.5E-07)
BVG96_22555	CUC46_17375	Lipoprotein precursor, NlpI	20.5 (2.1E-09)	8.5 (2.7E-05)
BVG96_23510	CUC46_23760	Peptide chain release factor 3	21.5 (<1.0E-10)	2.8 (<1.0E-10)

Table S2. *C. freundii* UMH14 mutant strains generated in this study.

Strain name	Relevant genotype	Description
tatC	Δ*tatC*::*nptII*	insertion of kanamycin resistance gene into 261-bp internal deletion of CfUMH14_4052/CUC46_20805 ORF
ruvA	Δ*ruvA*::*nptII*	insertion of kanamycin resistance gene into 429-bp internal deletion of CfUMH14_2031/CUC46_10495 ORF
nlpI	Δ*nlpI*::*nptII*	insertion of kanamycin resistance gene into 513-bp internal deletion of CfUMH14_3380/CUC46_17375 ORF
pepP	Δ*pepP*::*nptII*	insertion of kanamycin resistance gene into 729-bp internal deletion of CfUMH14_3123/CUC46_16060 ORF
fadR	Δ*fadR*::*nptII*	insertion of kanamycin resistance gene into 591-bp internal deletion of CfUMH14_1960/CUC46_10120 ORF
cysE	Δ*cysE*::*nptII*	insertion of kanamycin resistance gene into 723-bp internal deletion of CfUMH14_3802/CUC46_19530 ORF
znuB	Δ*znuB*::*nptII*	insertion of kanamycin resistance gene into 759-bp internal deletion of CfUMH14_2029/CUC46_10485 ORF
mtlD	Δ*mtlD*::*nptII*	insertion of kanamycin resistance gene into 1095-bp internal deletion of CfUMH14_3793/CUC46_19485 ORF
pfkA	Δ*pfkA*::*nptII*	insertion of kanamycin resistance gene into 936-bp internal deletion of CfUMH14_4131/CUC46_21195 ORF
sufI	Δ*sufI*::*nptII*	insertion of kanamycin resistance gene into 1392-bp internal deletion of CfUMH14_3219/CUC46_16565 ORF

Table S3. Oligonucleotide primers.

Name	Sequence (5′-3′)
Recombineering	
Δ*tatC*::kmF	gtacaggtatcaacggacattgccagctatctcagtttcgtcgtgtaggctggagctgcttc
Δ*tatC*::kmR	tttctcggtggcttcggcttcttttgcttcttcagcctcgttgtccatatgaatatcctccttagt
Δ*ruvA*::kmF	acccccgctggtccttctggaaacgggtggggtaggctatgaagtgcagtgtgtaggctggagctgcttc
Δ*ruvA*::kmR	gggcgggcaattttgctcaccatgcggctggcttcttgtggtttacatatgaatatcctccttagt
Δ*fadR*::kmF	atggtcattaaggcgcaaagcccggctgggttcgcggaagagtacatcattgtaggctggagctgcttcg
Δ*fadR*::kmR	cagattcttctgcatacgatgccaaatctcgccactgtcgtgaccgtagcatatgaatatcctccttagt
Δ*nlpI*::kmF	ttatatgagcgcggagtgttgtatgatagtctcggtctgagggcagtgtaggctggagctgcttc
Δ*nlpI*::kmR	aacgttgttagccaccgccagtttgaacaatgccgtagcgttgtccatatgaatatcctccttagt
Δ*cysE*::kmF	atgccgtgtgaagaactggatatcgtctggaaaaacattaaagcggaagctgtaggctggagctgcttcg
Δ*cysE*::kmR	tcagatgccatcgccgtattcgaaagtatgatgaatcccgttgaaatgccatatgaatatcctccttagt
Δ*znuB*::kmF	gatttacagggtcgtatcgtcttacgccggggaaatggtcactcatgatttgtaggctggagctgcttcg
Δ*znuB*::kmR	taagcgcagcgccaccgggcaacgcatgcttagctcgcttgctttttcatcatatgaatatcctccttagt
Δ*pfkA*::kmF	ccggcagcagatttcattttgcattccaaagttcagaggtagtcatgatttgtaggctggagctgcttcg
Δ*pfkA*::kmR	gcgaagcgccaccgggcaacaaaaaacaatcagtacagtttttcgcgcacatatgaatatcctccttagt
Δ*mtlD*::kmF	caccccagccctcttgggtaaaaacattgatgaaggttaatactatgaaatgtaggctggagctgcttcg
Δ*mtlD*::kmR	catcatttctttgcgttatacgcgttaacagcctcaagcacgacatcgctcatatgaatatcctccttagt
Δ*pepP*::kmF	cgcagcgcggacagtgaatacccgtaccgccaaagcagtgatttctggtacgtgtaggctggagctgcttc
Δ*pepP*::kmR	ggacgttccgggacgatacagacgcaggctggtctctaaggattccatatgaatatcctccttagt
Δ*sufI*::kmF	cgtgttaatagccagttttttatggagtttatatgtcattcagtcggcgttgtaggctggagctgcttcg
Δ*sufI*::kmR	cctgttctcgcgcatgttctggcgggcataacggaaatcccggcgcattacatatgaatatcctccttagt
Complementation	
*cysE*FOR	cttggcggcgcggtgaaaa
*cysE*REV	ccagttaggttatcccggcgcg
*mtlD*FOR	gatattgcccgcctggtgattg
*mtlD*REV	cagttctacggcggtgatgagg
*pepP*FOR	aagcttaagaacgaaacgcaaaggagagtg
*pepP*REV	ggatccgcgatcggggcagtgcaatgtgac
*pfkA*FOR	gcgcaagcttgaaccgggccagcattttgtgtat
*pfkA*REV	gcgctctagagttatcgcccgtttcctgcttcca
*sufI*FOR	gggaagccgccggtaaggttt
*sufI*REV	tggggctaggatttggcgtcac
*tatC*FOR	aagcttcgttgatggatgcagaaaagaaag
*tatC*REV	gaattcctcccatatgacaaccgccctgac
Site-directed mutagenesis	
pepPflagFOR	gattacaaggatgacgacgataagtgagcgtgattatcgttggtgg
pepPflagREV	cttatcgtcgtcatccttgtaatcttgcagtcgcgccgcagcc
sufIflagFOR	gattacaaggatgacgacgataagtaatgcgccgggatttccgtta
sufIflagFOR	cttatcgtcgtcatccttgtaatccggcgccgggttcaccaac

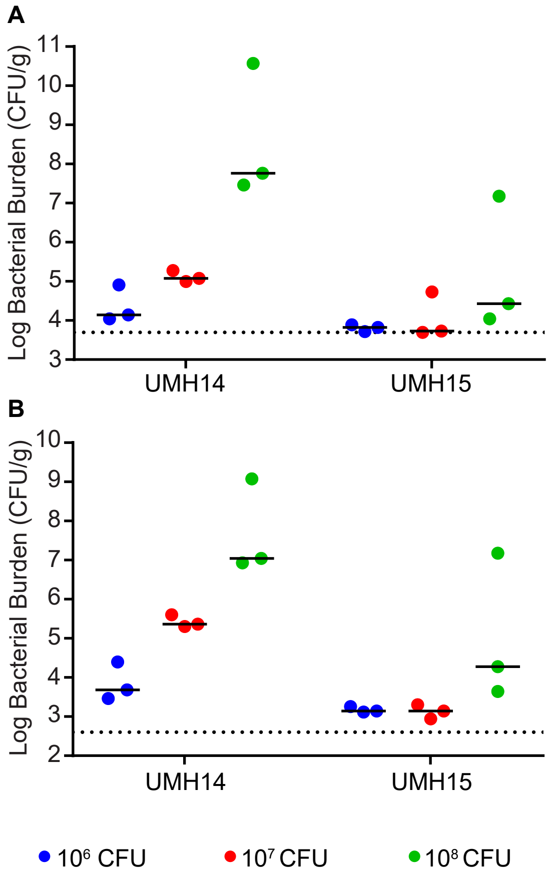

Figure S1. Recovery of *C. freundii* from mice following infection at different doses. Mice were inoculated with *C. freundii* strains UMH14 or UMH15 via tail vein injection at the indicated target doses. The bacterial burden in spleen (A) and liver (B) was determined after 24 hours from organ homogenates. Solid horizontal lines indicate the median bacterial burden for each strain and the dotted line represents the limit of detection.

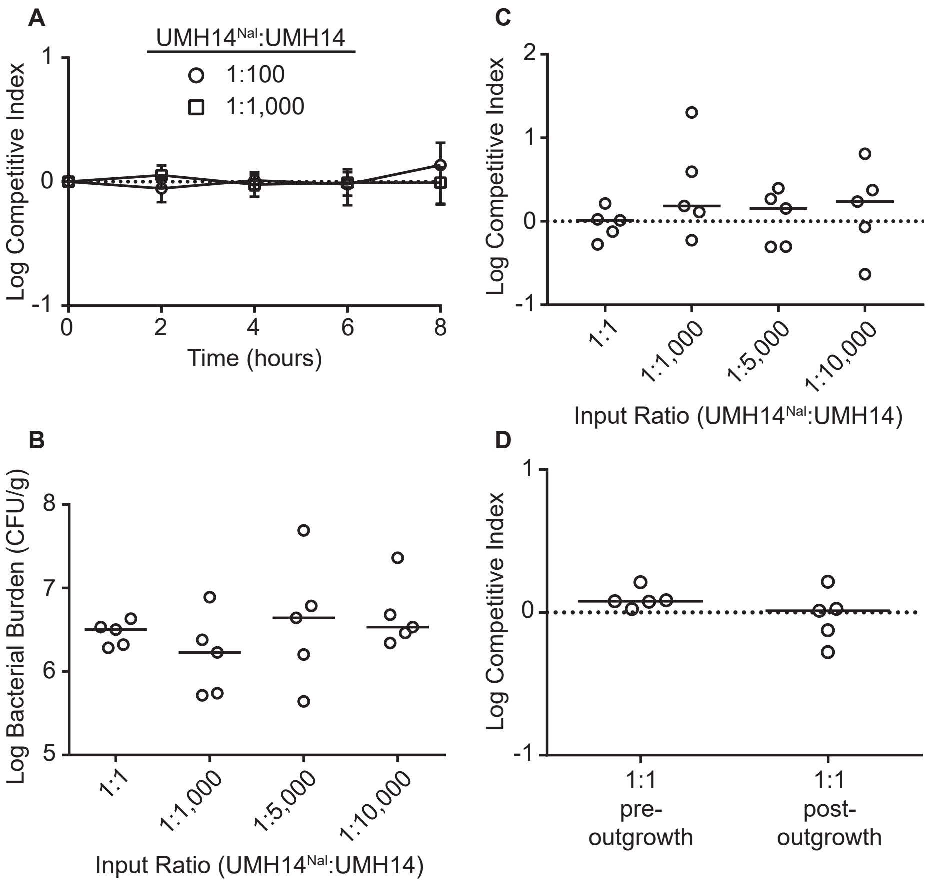

Figure S2. Assessment of potential colonization bottlenecks in the bacteremia model. A. *C. freundii* strains UMH14^{Nal} and UMH14 were inoculated into LB medium at the indicated ratios. The abundance of each strain was determined by viable count and used to calculate the CI relative to the time zero ratio. Data represent the mean ± standard deviation from triplicate cultures. None of the values differed significantly from the hypothesized value of 1.0 (dotted line) by one-sample *t*-test ($P>0.05$). B. Mice were infected with mixtures of UMH14^{Nal} and UMH14 at the indicated ratios. The total bacterial burden from each mouse as determined by viable count from spleen homogenates is shown with horizontal lines representing the median. C. The input and output ratio of the two *C. freundii* strains recovered from mice infected in panel B was used to determine the CI after outgrowth of spleen homogenates in LB medium for five hours. None of the median CI values were significantly different from the theoretical value of 1.0 (dotted line) as determined by Wilcoxon signed rank test ($P>0.05$). D. The CI for bacteria recovered from spleen homogenates containing the 1:1 UMH14^{Nal}:UMH14 infection mixture was determined directly (pre-outgrowth) or after outgrowth in LB medium (post-outgrowth) as described for panel C. The median values were not statistically different between the two methods using the Mann-Whitney test ($P>0.05$).

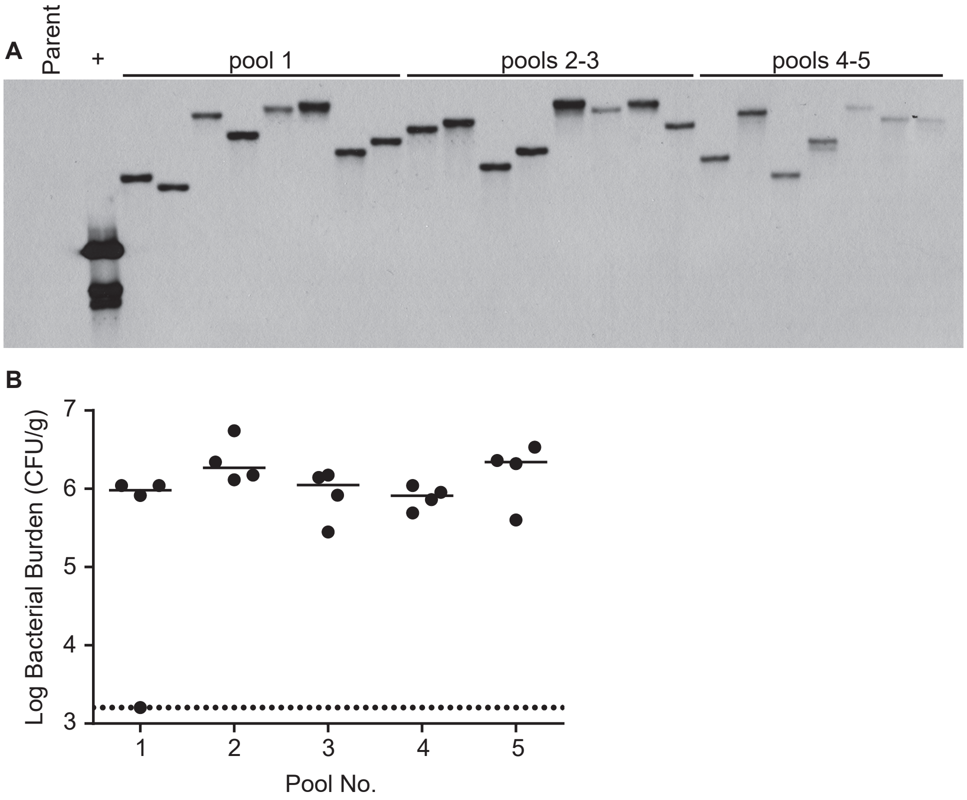

Figure S3. Random transposon insertion into the *C. freundii* genome and mouse infections with the mutant library. A. UMH14 transposon insertion mutants were selected randomly from the mating pools and HindIII-digested genomic DNA from each isolate was probed for hybridization to an internal fragment of the transposon-encoded kanamycin resistance gene. Genomic DNA from the UMH14 parent strain was included as a negative control and pSAM_Cm plasmid DNA was included as a positive control (+). B. Total splenic bacterial burden of mice infected with the five transposon mutant pools 24 hours after inoculation. Medians are indicated by horizontal lines and the limit of detection is represented by the dotted line.

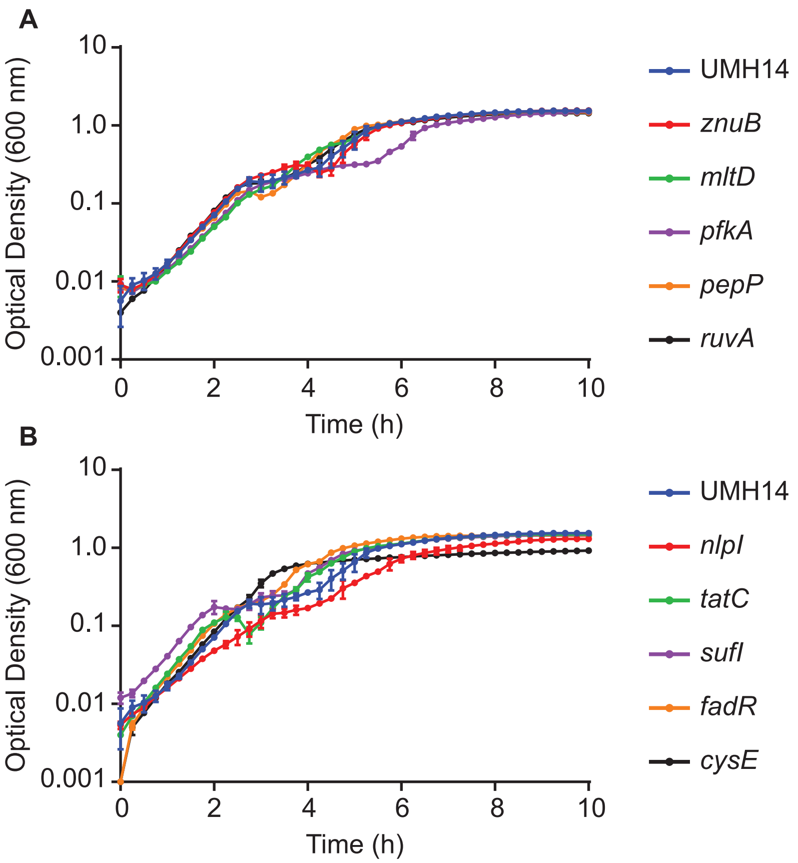

Figure S4. Growth of *C. freundii* mutants in rich medium. Parent strain UMH14 and mutant derivatives were cultured in LB medium. Growth was measured by optical density (600 nm) at 15-minute intervals and the mean from triplicate cultures ± standard deviation is shown. Mutant strain growth curves were arbitrarily distributed between panels A and B.

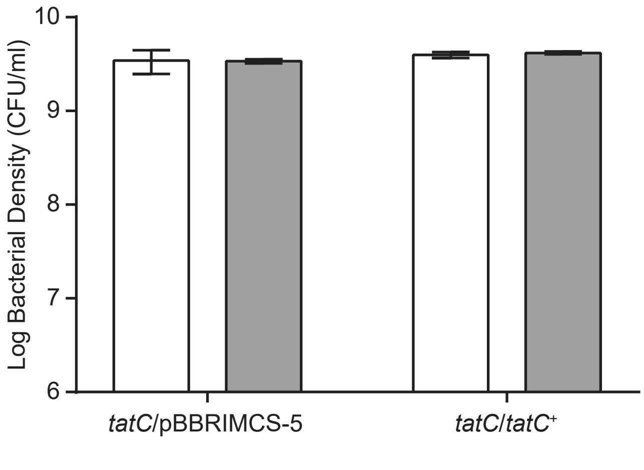

Figure S5. Stable replication of pBBR1MCS-5 plasmids in the absence of selection. The *C. freundii* UMH14 *tatC* mutant harboring either the pBBR1MCS-5 empty vector or the *tatC*+ complementation plasmid was propagated in LB medium in the absence of antibiotic selection (open bars) or in the presence of gentamicin (shaded bars) for 24 hours. The number of viable bacteria retaining plasmids was then determined for both culture conditions by plating on agar medium containing gentamicin. Error bars indicate the standard deviation from triplicate cultures. The number of gentamicin-resistant viable bacteria was not significantly different between the two culture conditions as determined by *t*-test (P>0.05).

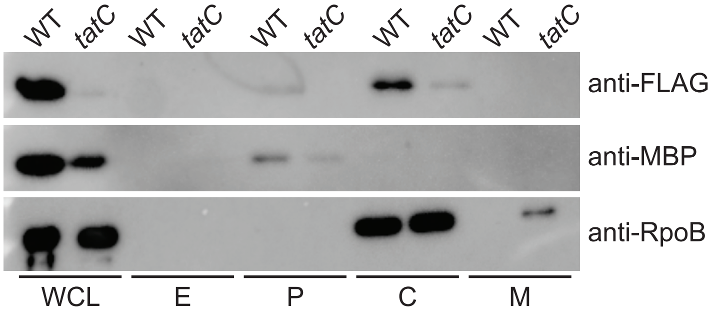

Figure S6. Immunoblots of subcellular fractions from bacteria producing the PepP^{FLAG} protein. Wild-type and *tatC* mutant bacteria were fractionated into extracellular (E), periplasmic (P), cytoplasmic (C), and crude membrane (M) preparations. Proteins from each fraction and whole-cell lysate (WCL) controls were separated by SDS-PAGE and immunoblotting was performed using the indicated antibodies. The anti-MBP and anti-RpoB antibodies were used as a control for the periplasmic and cytoplasmic fractions, respectively. Images from independent blots are separated by white space and were cropped to show only the relevant segment.
